# Supplementary material for: Genome-Wide Identification of PAP1 Direct Targets in Regulating Seed Anthocyanin Biosynthesis in Arabidopsis
Source: Int J Mol Sci. 2023 Nov 7;24(22):16049. doi: 10.3390/ijms242216049 (PMC10671800; doi:10.3390/ijms242216049)
Supplement: Supplementary file 1 [file ijms-24-16049-s001.zip › ijms-2646898-supplementary/Supplementary figures and tables/Supplementary figure.pdf]

Supplementary Figure

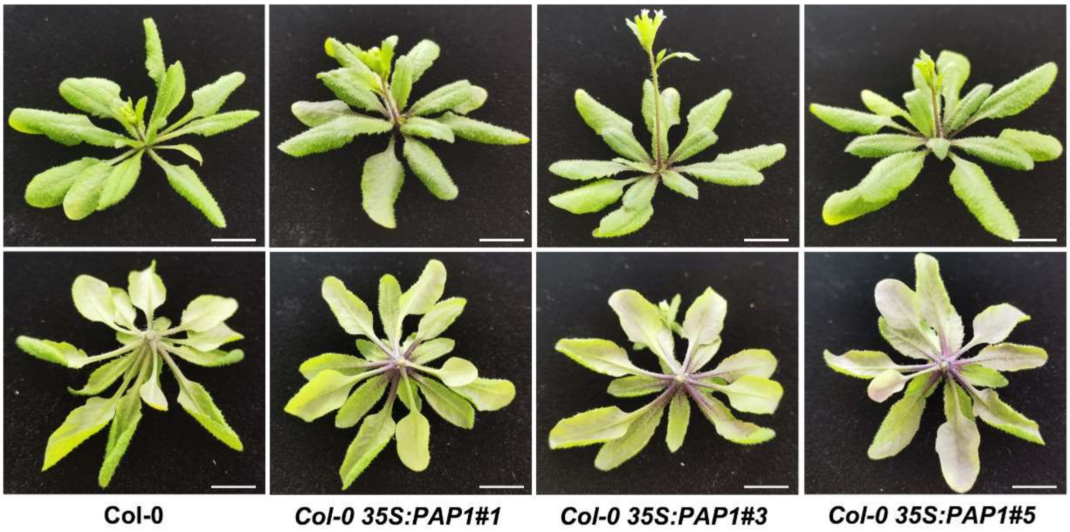

Supplementary Figure S1. Phenotypes of 30 days old wild-type (Col-0) and three T_3 homozygous transgenic plants overexpressing the *PAP1* gene (*Col-0 35S:PAP1*). Bars = 1 cm.
